# Efficacy of artesunate-amodiaquine and artemether-lumefantrine for uncomplicated *Plasmodium falciparum* malaria in Madagascar, 2018

**DOI:** 10.1186/s12936-021-03935-4

**Published:** 2021-11-03

**Authors:** Catherine M. Dentinger, Tovonahary Angelo Rakotomanga, Antsa Rakotondrandriana, Arinomenjanahary Rakotoarisoa, Marie Ange Rason, Leah F. Moriarty, Laura C. Steinhardt, Laurent Kapesa, Jocelyn Razafindrakoto, Samaly S. Svigel, Naomi W. Lucchi, Venkatachalam Udhayakumar, Eric S. Halsey, C. Arsène Ratsimbasoa

**Affiliations:** 1 Malaria Branch, Division of Parasitic Diseases and Malaria, Center for Global Health, US Centers for Disease Control and Prevention, Atlanta, Georgia; 2 US President’s Malaria Initiative, US Centers for Disease Control and Prevention, Antananarivo, Madagascar; 3 National Malaria Control Programme, Ministry of Health, Antananarivo, Madagascar; 4 University of Antananarivo, Antananarivo, Madagascar; 5 US President’s Malaria Initiative, US Centers for Disease Control and Prevention, Atlanta, Georgia; 6 US President’s Malaria Initiative, USAID, Antananarivo, Madagascar; 7 University of Fianarantsoa, Fianarantsoa, Madagascar; 8 Centre National d’ Application de Recherche Pharmaceutique, Antananarivo, Madagascar

**Keywords:** Madagascar, Anti-malarial, Artemisinin-based combination therapy, Resistance, Efficacy

## Abstract

**Background:**

Since 2005, artemisinin-based combination therapy (ACT) has been recommended to treat uncomplicated falciparum malaria in Madagascar. Artesunate-amodiaquine (ASAQ) and artemether-lumefantrine (AL) are the first- and second-line treatments, respectively. A therapeutic efficacy study was conducted to assess ACT efficacy and molecular markers of anti-malarial resistance.

**Methods:**

Children aged six months to 14 years with uncomplicated falciparum malaria and a parasitaemia of 1000–100,000 parasites/µl determined by microscopy were enrolled from May–September 2018 in a 28-day in vivo trial using the 2009 World Health Organization protocol for monitoring anti-malarial efficacy. Participants from two communes, Ankazomborona (tropical, northwest) and Matanga (equatorial, southeast), were randomly assigned to ASAQ or AL arms at their respective sites. PCR correction was achieved by genotyping seven neutral microsatellites in paired pre- and post-treatment samples. Genotyping assays for molecular markers of resistance in the *pfk13*, *pfcrt* and *pfmdr1* genes were conducted.

**Results:**

Of 344 patients enrolled, 167/172 (97%) receiving ASAQ and 168/172 (98%) receiving AL completed the study. For ASAQ, the day-28 cumulative PCR-uncorrected efficacy was 100% (95% CI 100–100) and 95% (95% CI 91–100) for Ankazomborona and Matanga, respectively; for AL, it was 99% (95% CI 97–100) in Ankazomborona and 83% (95% CI 76–92) in Matanga. The day-28 cumulative PCR-corrected efficacy for ASAQ was 100% (95% CI 100–100) and 98% (95% CI 95–100) for Ankazomborona and Matanga, respectively; for AL, it was 100% (95% CI 99–100) in Ankazomborona and 95% (95% CI 91–100) in Matanga. Of 83 successfully sequenced samples for *pfk13*, no mutation associated with artemisinin resistance was observed. A majority of successfully sequenced samples for *pfmdr1* carried either the NFD or NYD haplotypes corresponding to codons 86, 184 and 1246. Of 82 successfully sequenced samples for *pfcrt*, all were wild type at codons 72–76.

**Conclusion:**

PCR-corrected analysis indicated that ASAQ and AL have therapeutic efficacies above the 90% WHO acceptable cut-off. No genetic evidence of resistance to artemisinin was observed, which is consistent with the clinical outcome data. However, the most common *pfmdr1* haplotypes were NYD and NFD, previously associated with tolerance to lumefantrine.

**Supplementary Information:**

The online version contains supplementary material available at 10.1186/s12936-021-03935-4.

## Background

Malaria remains a public health problem in Madagascar, an Indian Ocean island-nation with a population of 25.6 million [1]. In 2019, malaria was the fourth leading cause of death, and over one million cases of disease were reported nationally [2]. Although malaria is endemic throughout the country, prevalence is heterogeneous; rates of disease are lowest in the central highlands and highest along the east and west coasts of the island. According to routine public health surveillance data, this heterogeneity has increased in recent years, with some areas advancing toward malaria elimination and others experiencing upsurges and focal outbreaks of disease. Since 2005, the National Malaria Control Programme (NMCP) has recommended artemisinin-based combination therapy (ACT) for treatment of confirmed uncomplicated falciparum malaria [3]. In Madagascar, artesunate-amodiaquine (ASAQ) and artemether-lumefantrine (AL) are the first- and second-line treatment recommendations, respectively.

Resistance to artemisinin-based drugs has been reported in Asia [4–6], and the threat of resistance to these drugs in Madagascar is of concern. Evidence of partial resistance to artemisinin compounds was recently reported in sub-Saharan Africa (SSA), amplifying these concerns [7].

The World Health Organization (WHO) recommends that malaria control programmes conduct periodic studies to monitor the therapeutic efficacy of recommended anti-malarials and to identify early emergence of resistance markers [8]. The most recent therapeutic efficacy study (TES) was conducted in 2016 in two Madagascar districts, Ifanadiana and Maevatanana, to monitor ASAQ. Results indicated that ASAQ had acceptable clinical efficacy with a 100% (95% CI 97–100) polymerase chain reaction (PCR)-corrected day-28 cure rate at both study sites [9]. For AL, however, no recent studies to evaluate therapeutic efficacy have been done in Madagascar. Evidence of reduced AL efficacy has been described in SSA [10–12]; given that it is the second-line anti-malarial in Madagascar, and has at times been used as the first-line treatment due to shortages of ASAQ, describing its efficacy in Madagascar is important.

In accordance with WHO recommendations for anti-malarial drug monitoring, the NMCP and partners conducted a TES in 2018 to evaluate the efficacy of the first- and second-line anti-malarials. In addition, *Plasmodium falciparum* parasite specimens were tested for the presence of molecular markers associated with resistance or reduced susceptibility to artemisinin, amodiaquine and lumefantrine [13–17]. The results of this TES are described in this paper and the implications of the findings are discussed.

## Methods

### Study setting and design

**Fig. 1:**
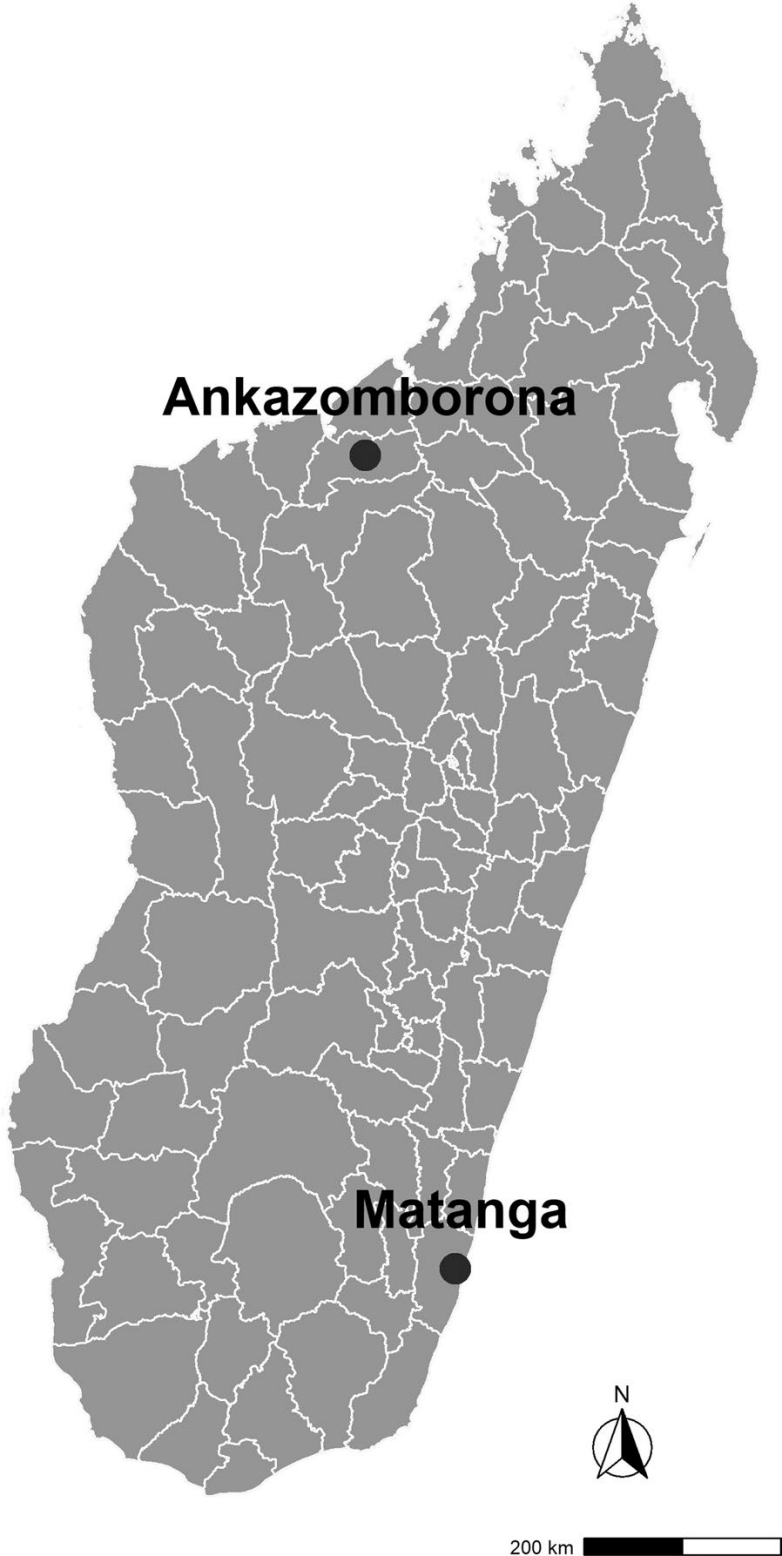
Map of Madagascar with 2018 National Malaria Control Program Therapeutic Efficacy Study sites

An in vivo open-label study to test the efficacy and tolerance of ASAQ and AL for treatment of laboratory-confirmed uncomplicated falciparum malaria among Malagasy children was based on the standard WHO protocol [8]. Cases were recruited during May–September 2018 in two basic health centres (*Centre de Santé de Base* (CSB)) serving the communes of Ankazomborona, in Marovoay District in the northwest, and Matanga, in Vangaindrano District in the southeast (Fig. 1). These CSBs were selected to represent different climatic zones of the country; Ankazomborona is in the tropical zone and Matanga is in the equatorial zone. Both communes are in districts with moderate malaria transmission, defined as 50–100 cases per 1000 population per year [3]; in 2016, malaria parasite prevalence by microscopy among children 6–59 months of age was 9.0 and 8.8% in the zones encompassing Ankazomborona and Matanga, respectively [18]. Children in each site were randomly assigned to receive either ASAQ (Winthrop®, Sanofi-Aventis, France) or AL (Coartem®, Novartis, Basel, Switzerland) and treated according to WHO/NMCP recommendations. To ensure study quality, staff from the US President’s Malaria Initiative (PMI) and the NMCP visited each site to observe study procedures and data management and provide feedback and support.

### Primary and secondary endpoints and sample size

The primary endpoints were 28-day uncorrected and PCR-corrected efficacy. Sample size calculations were based on 28-day PCR-corrected efficacy and powered assuming a 5% failure rate with a 5% margin of error and 95% confidence level. Assuming loss to follow-up of 15%, a minimum of 86 patients per arm was needed at each site, for a total of 344 patients across the two sites. Secondary endpoints included early treatment failures, day-3 parasite clearance, late clinical and parasitological failures, and the presence of single nucleotide polymorphisms (SNPs) associated with anti-malarial resistance or decreased response in the *pfk13, pfmdr-1* and *pfcrt* genes.

### Study population and participant enrolment

All children aged six months to 14 years presenting to the CSB for evaluation of febrile illness were referred to the TES team, who explained the study to parents. A rapid diagnostic test (RDT) (SD Bioline Malaria Ag *Pf*/Pan, Standard Diagnostics, Inc.) was administered according to the manufacturer’s instructions to diagnose malaria, and thick and thin smears for microscopy were prepared. All children with a positive RDT for *P. falciparum*, that was confirmed by microscopy to be *P. falciparum* mono-infection with a density of 1000–100,000 parasites/µl of blood, were invited to participate. Children with a negative RDT, and those without parental consent or assent (for those aged 7 to 14 years), were referred to the CSB clinician for care. After consent, a questionnaire was administered to eligible children or parents to collect demographic and clinical information and a physical examination was performed by the study physician. Pregnancy and lactation were assessed for all females ≥ 12 years of age; for those who had attained menarche and missed a menstrual period, a pregnancy test was done. Pregnant or lactating females were excluded and referred to the CSB clinician for care. A second finger stick was done to measure haemoglobin concentration (Hemocue®, HB 201 + , Angelholm, Sweden); children with haemoglobin concentration < 8 g/dl were excluded and referred to the CSB clinician. Capillary blood for dried blood spots (DBS) was also collected on Whatman 903 filter papers (GE Healthcare Life Sciences, PA, USA) during enrolment and used for molecular analyses. Additional criteria for enrolment included weight ≥ 5 kg, ability to take oral medication, and plans to remain in the study area for the following 28 days. Exclusion criteria included: signs of severe disease (i.e., prostration, change in mental status, convulsions, respiratory distress, persistent vomiting, haemoglobinuria, jaundice, haemorrhagic shock); underlying chronic illness or signs of severe malnutrition; reported allergy to one of the ACT; having taken an anti-malarial medication within the previous 30 days; having taken a medication which could interfere with one of the study medications; participation in another clinical study; and, residence > 15 km from the CSB. Enrolled children were randomly assigned to treatment with either AL or ASAQ using block randomization, by age group (6–59 months, 5–9 years, 10–14 years), and a one-to-one ratio for the two medications. Once a child was enrolled, study staff selected the top envelope for that child’s age group, which held the treatment indication.

### Participant treatment, monitoring and follow-up

Children were treated by study personnel with a full course of either ASAQ or AL (tablets were crushed with water as needed) on days 0, 1 and 2 according to WHO/NMCP recommendations. For ASAQ, the following doses were administered based on the child’s weight: 5 to < 9 kg, 25 mg artesunate + 67.5 mg amodiaquine base; 9 to  < 18 kg, 50 mg artesunate + 135 mg amodiaquine base; 18 to  < 36 kg, 100 mg artesunate + 270 mg amodiaquine base; ≥ 36 kg, 200 mg artesunate + 540 mg amodiaquine base. For AL, a first dose was given at time 0 followed by a second dose 8 h later; on days 2 and 3, the child was treated twice per day. Medication dose was based on the child’s weight: 5–15 kg, 20 mg artemether + 120 mg lumefantrine; 15 to  < 25 kg, 40 mg artemether + 240 mg lumefantrine; 25 to  < 35 kg, 60 mg artemether + 360 mg lumefantrine; ≥ 35 kg, 80 mg artemether + 480 mg lumefantrine. All doses of medication, including the evening dose of AL, were administered with water only and were directly observed and monitored by study team personnel. Clinical and parasitological response to treatment and screening for adverse drug events occurred on days 1, 2, 3, 7, 14, 21, and 28; parents were instructed to bring their child to the health centre if symptoms occurred between scheduled visits. Blood samples, for microscopy and preparation of DBS, were collected from a finger stick at every visit, including unscheduled visits. Participants were withdrawn from the study and referred for care if they developed signs of severe disease or if an adverse event required discontinuing study treatment.

Study participants were classified as early treatment failures in the following situations: signs of severe malaria in the presence of parasitaemia up to day-3; day-2 parasitaemia higher than at day-0; day-3 parasitaemia above 25% of day-0 parasitaemia in the absence of fever; parasitaemia detected on day-3 in the presence of fever. Participants were classified as late treatment failures if they had not demonstrated early treatment failure and had at least one blood slide positive for asexual *P. falciparum* parasites after day-3. Patients negative for malaria on day-28 and not having previously met any treatment failure criteria, were classified as adequate clinical and parasitological response (ACPR). Participants with early or late treatment failure were discontinued and referred for care.

### Microscopy

Microscopy slides were prepared at the study sites according to WHO guidelines; the first slide was prepared by staining with 10% Giemsa for 10–15 min to confirm the diagnosis and facilitate treatment. All slides were read on site by WHO certified level 1 or 2 microscopists to confirm mono-infection and calculate parasite density. Parasite density, expressed as the number of asexual parasites per µl of blood, was calculated by dividing the number of asexual parasites by the number of white blood cells and then multiplying by an assumed white blood cell density of 8000 per by study personnel. Slides were considered negative if no parasites were seen after examination of 200 oil-immersion fields in a thick blood film. Slides were read by two microscopists; for parasite density, the mean of the two readers was reported. Discrepant results (> = 25%) were resolved by a third certified reader. For parasite density discrepancies ≥ 25%, the mean value across all three readers was reported. To ensure microscopy quality, 20% of slides were randomly selected for re-reading after the study by WHO-certified NMCP microscopists, who were blinded to the original result.

### DNA extraction and molecular analysis

Collected DBS were transported to the NMCP malaria reference laboratory in Antananarivo, the capital of Madagascar, where parasite DNA was extracted from DBS with QIAamp 96 DNA Blood kit (No. 51306) according to the manufacturer’s instructions (Quiagen Inc., Hilden, Germany).

Molecular analyses were performed on the Madagascar specimens by Madagascar laboratorians at the US Centers for Disease Control and Prevention Malaria Laboratory in Atlanta, GA, as part of the technical training objective of the PMI-sponsored Antimalarial Resistance Monitoring in Africa (PARMA) network [19]. Investigation of polymorphisms in codons 389–638 of the *pfk13* propeller domain, codons 86, 184, 1034, 1042, and 1246 of *pfmdr1,* and codons 72–76 of *pfcrt* were done using Sanger sequencing [20] on 85 day-0 and 18 day-of-recurrent-parasitaemia samples. The analysis of SNPs was done using the Geneious software package (Biomatters Inc., San Francisco, CA, USA) utilizing the 3D7 *pfk13* (PF3D7_1343700), *pfmdr1* (PF3D7_0523000) and *pfcrt* sequences (PF3D7_0709000) as references. Raw sequence reads were cleaned using default settings and reads with high-quality scores (> 30%) were further analysed.

### Differentiation between recrudescence and re-infection

PCR correction, to differentiate recrudescence from re-infection in those with a late treatment failure, was achieved by comparing seven neutral microsatellite genotypes (TA1 on chromosome 6, Poly-α on chromosome 4, PfPK2 on chromosome 12, 2490 on chromosome 10, C2M34-313 on chromosome 2, C2M69-383 on chromosome 3, and TA109 on chromosome 6) in the paired pre-treatment and post-treatment samples using previously described methods [21, 22]. The sizes of the amplification products were determined by capillary electrophoresis on an Applied Biosystems 3130 xl sequencer (Applied Biosystems, Foster City, CA, USA). A previously validated Bayesian algorithm was used to generate a posterior probability of recrudescence for each late treatment failure [23].

### Statistical analysis

Data were recorded in the field into WHO-standard templates [24] and later double-entered into an Excel database at the NMCP’s reference laboratory. Statistical analyses were performed using R version 4.0.1 (R Foundation for Statistical Computing, Vienna, Austria).

Uncorrected and PCR-corrected per protocol efficacy for each site and drug was calculated by dividing the number of participants classified as ACPR over all participants reaching a study outcome. The sum of posterior probabilities of recrudescence was used to calculate the total number of recrudescent infections for the PCR-corrected analyses. Re-infections were removed from the calculations of PCR-corrected per protocol efficacy. For Kaplan–Meier cumulative efficacy estimates, re-infections were removed (censored) on the day of re-infection and participants lost to follow-up or who withdrew were included until the last day of follow-up in uncorrected and PCR-corrected analysis. Posterior sampling was used to generate the PCR-corrected Kaplan–Meier estimates and 95% confidence intervals using the posterior probabilities of recrudescence.

### Ethical considerations

The study was reviewed and approved by the institutional Ethics Committee of Biomedical Research of the Ministry of Public Health of Madagascar. The US CDC’s Center for Global Health Office of the Associate Director for Science determined CDC staff to be non-engaged in this research study (CDC human subjects 2016-012a). Local leaders and community members were informed of the study through meetings and radio broadcasts. Participants and parents were informed about the objectives of the project, benefits and risks associated with participation in the study; signed informed consent was obtained from parents before enrolment and children aged 7–14 years provided verbal assent.

## Results

### Study population

**Fig. 2:**
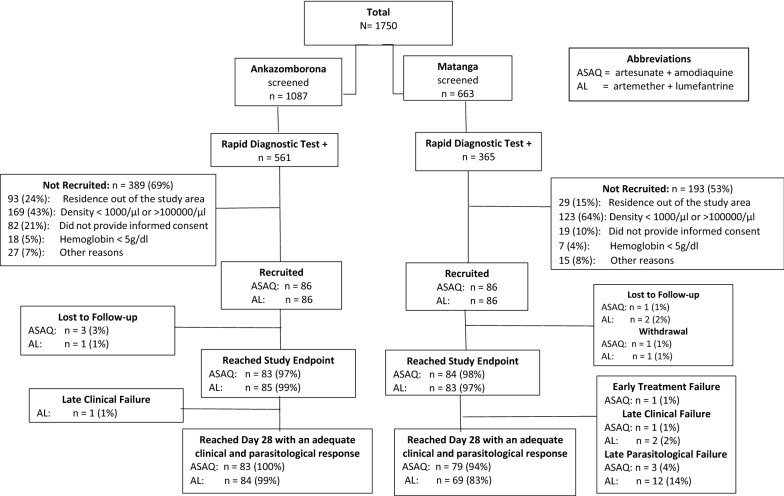
Participant disposition, antimalarial therapeutic efficacy study, Madagascar 2018

Of 1087 children in Ankazomborona and 663 Matanga who presented to the study site with fever, 561 and 365 were RDT-positive, respectively. Of these, 172 children, 86 in each treatment arm, met the study inclusion criteria and were enrolled at each study site. A total of 168 (83 for ASAQ and 85 for AL) and 167 (84 for ASAQ and 83 for AL) children reached the study endpoint in accordance with the study protocol in Ankazomborona and Matanga, respectively (Fig. 2). Demographic characteristics and parasite concentration of the enrolled children are reported in Table 1.

**Table 1:** Participants’ characteristics at enrollment, Ankazomborona and Matanga Districts, Madagascar, 2018 (n = 344)

	Ankazomborona		Matanga	
	ASAQ (n = 86)	AL (n = 86)	ASAQ (n = 86)	AL (n = 86)
Age (months), median (range)	100 (19–180)	108 (18–178)	72 (8–180)	60 (8–180)
Female, n (%)	37 (43)	34 (40)	41 (48)	42 (49)
Weight (kg), mean, sd	21 (8)	21 (8)	18 (7)	18 (8)
Baseline parasitemia, median parasites/µL (range)	22,293 (1007–88,492)	22,249 (1162–93,915)	11,507 (1093–91,264)	17,445 (1036–94,717)
Gametocytemia on day 0, n (%)	3 (3)	3 (3)	4 (5)	2 (2)
Reached Study end point, n (%)	83 (97)	85 (99)	84 (98)	83 (97)

*ASAQ* artesunate-amodiaquine, *AL* artemether-lumefantrine, *sd* standard deviation

### Efficacy

The uncorrected cumulative efficacy of ASAQ was 100% (95% CI 100–100) in Ankazomborona and 95% (95% CI 91–100) in Matanga. The PCR-corrected cumulative efficacy of ASAQ was 100% (95% CI 100–100) in Ankazomborona and 98% (95% CI 95–100) in Matanga (classification by genotyping can be found in Additional file 1: Table S1). In the ASAQ arm, one patient was parasitaemic on day-3 in Matanga and was classified as an early treatment failure. There were no late treatment failures observed in Ankazomborona. In Matanga, in addition to the early treatment failure, there was one late clinical failure, and three late parasitological failures in the ASAQ arm.

The uncorrected cumulative efficacy of AL was 99% (95% CI 97–100) in Ankazomborona and 83% (95% CI 76–92) in Matanga (classification by genotyping can be found in Additional file 1: Table S1). The PCR-corrected cumulative efficacy of AL was 100% (95% CI 99–100) in Ankazomborona and 95% (95% CI 91–100) in Matanga. In the AL arm, there were no patients with parasitaemia on day-3 in Ankazomborona. There was one child in the AL arm in the Matanga site who had 14 parasites per µl on day-3 which had cleared by day-7. There was one late clinical failure in the Ankazomborona AL arm. There were two (2%) late clinical failures and 12 (14%) late parasitological failures in Matanga in the AL arm (see Table 2).

**Table 2:** 28-day uncorrected and PCR-corrected efficacy estimate, by drug, by site, Madagascar, 2018

	Ankazomborona		Matanga	
	ASAQ n = 83	AL n = 85	ASAQ n = 84	AL n = 83
Parasitemia day 2, n (%)	1 (1)	14 (16)	8 (10)	9 (10)
Parasitemia day 3, n (%)	0	0	1 (1)	1 (1)
Early treatment failure, n (%)	0	0	1	0
Late clinical failure, n (%)	0	1 (1)	1 (1)	2 (2)
Late parasitological failure, n (%)	0	0	3 (4)	12 (14)^c^
Recrudescence^a^	0	0	2	2
Day 14–21	0	0	1	0
Day 22–28	0	0	1	2
Reinfection	0	1	2	11
Day 14–21	0	0	0	3
Day 22–28	0	1	2	8
Per protocol efficacy, n (% [95% CI]), uncorrected	83 (100 [96–100])	84 (99 [94–100)	79 (94 [87–98])	69 (83 [73–90])
Per protocol efficacy, n (% [95% CI]), PCR-corrected^b^	83 (100 [96–100])	84 (100 [96–100])	79 (97 [90–100])	69 (95 [87–99])
Kaplan–Meier estimate of efficacy, uncorrected (% [95% CI])	100 (100–100)	99 (97–100)	95 (91–100)	83 (76–92)
Kaplan–Meier estimate of efficacy, PCR-corrected (% [95% CI])^a^	100 (100–100)	100 (99–100)	98 (95–100)	95 (91–100)

*ASAQ* artesunate-amodiaquine, *AL* artemether-lumefantrine, *CI* confidence interval

^a^Posterior probability of recrudescence ≥ 0.5

^b^Posterior probability of recrudescence rather than whole numbers used for PCR-corrected estimates

^**c**^One late treatment failure had an indeterminate PCR

### Molecular markers of resistance

A total of 85 day-0 and 18 day-of-recurrent-parasitaemia samples were assessed for presence of SNPs in *pfk13, pfmdr1* and *pfcrt.* Of these, 83/85 (98%) day-0 and 14/18 (78%) day of recurrent parasitaemia samples were successfully sequenced for *pfk13* and were all found to be wild type at the investigated codons (Table 3). For *pfmdr1*, mutations were found only in codons 86, 184 and 1246 (Tables 3 and 4). All samples were wild type for 1034 and 1042. *pfmdr1* haplotypes were constructed using codons N86Y, Y184F and D1246Y. There were 74/85 (87%) day-0 and 14/18 (78%) day of recurrent parasitaemia samples successfully sequenced at these three codons. The NFD and NYD haplotypes were found in 37 (50%) and 30 (41%) of all day-0 samples, respectively. For *pfcrt* codons 72–76, no mutations were found in the 82/85 day-0 (95%) and 13/18 (72%) day of recurrent parasitaemia samples that were successfully amplified.

**Table 3:** Prevalence of polymorphisms from pre-treatment samples observed during a therapeutic efficacy study in Madagascar, stratified by eventual outcome

	Overall N = 85	ACPR N = 65	ETF N = 1	Recrudescence N = 4	Reinfection N = 14
*pfk13*^a^	n = 83	n = 65	n = 1	n = 3	n = 14
Wild type (no mutations detected)	83 (100%)	65 (100%)	1 (100%)	3 (100%)	14 (100%)
*pfmdr1 *N86**Y**					
Successfully amplified	n = 81	n = 62	n = 1	n = 4	n = 14
N	64 (79%)	48 (78%)	1 (100%)	3 (75%)	12 (86%)
N/Y	2 (3%)	2 (3%)	0 (0%)	0 (0%)	0 (0%)
Y	15 (19%)	12 (19%)	0 (0%)	1 (25%)	2 (14%)
Y184**F**					
Successfully amplified	n = 82	n = 63	n = 1	n = 4	n = 14
Y	34 (41%)	23 (36%)	1 (100%)	1 (25%)	9 (64%)
Y/F	7 (9)	6 (10%)	0 (0%)	0 (0%)	1 (7%)
F	41 (50%)	34 (54%)	0 (0%)	3 (75%)	4 (29%)
S1034**C**					
Successfully amplified	n = 77	n = 59	n = 1	n = 4	n = 13
S	77 (100%)	59 (100%)	1 (100%)	4 (100%)	13 (100%)
N1042**D**					
Successfully amplified	n = 77	n = 59	n = 1	n = 4	n = 13
N	77 (100%)	59 (100%)	1 (100%)	4 (100%)	13 (100%)
D1246**Y**					
Successfully amplified	n = 77	n = 59	n = 1	n = 4	n = 13
D	73 (95%)	57 (97%)	1 (100%)	3 (75%)	12 (92%)
D/Y	0 (0%)	0 (0%)	0 (0%)	0 (0%)	0 (0%)
Y	4 (5%)	2 (3%)	0 (0%)	1 (25%)	1 (8%)
*pfmdr1* haplotypes^b^					
Amplified for all 3 codons (86, 184, 1246)	n = 74	n = 56	n = 1	n = 4	n = 13
NFD	37 (50%)	28 (50%)	1 (100%)	3 (75%)	5 (27%)
NYD	30 (41%)	23 (41%)	0 (0%)	0 (0%)	7 (38%)
YFD	6 (8%)	5 (9%)	0 (0%)	1 (25%)	0 (0%)
YYD	8 (11%)	6 (11%)	0 (0%)	0 (0%)	2 (11%)
YYY	1 (1%)	0 (0%)	0 (0%)	0 (0%)	1 (5%)
YFY	1 (1%)	1 (1%)	0 (0%)	0 (0%)	0 (0%)
NFY	2 (3%)	1 (1%)	0 (0%)	0 (0%)	1 (5%)
*pfcrt* haplotypes					
Amplified for all 5 codons (72—76)	n = 82	n = 63	n = 1	n = 4	n = 14
CVMNK	82 (100%)	63 (100%)	1 (100%)	4 (100%)	14 (100%)

*ACPR* adequate clinical and parasitological response, *ETF* early treatment failure

^a^Investigated SNPs: F446I, N456Y, M476I, Y493H, R539T, I543T, P553L, R561H, C580Y

^b^mixed infections included in the numerator for each haplotype; therefore, column totals may sum to > 100%

**Table 4:** Prevalence of *pfmdr1* polymorphisms in pre-treatment and post-treatment samples stratified by site and treatment arms

	Ankazomborona				Matanga			
	Pre-treatment		Post-treatment		Pre-treatment		Post-treatment	
	AL n (%)	ASAQ n (%)	AL n (%)	ASAQ n (%)	AL n (%)	ASAQ n (%)	AL n (%)	ASAQ n (%)
	N = 15	N = 16	N = 1	N = 0	N = 29	N = 21	N = 10	N = 3
SNPs, codon 86								
N86	10 (67)	12 (75)	1 (100)	(0) 0	26 (90)	16 (76)	9 (90)	3 (100)
86 N/Y	0 (0)	0 (0)	0 (0)	0 (0)	0 (0)	2 (10)	0 (0)	0 (0)
86Y	5 (33)	4 (25)	0 (0)	0 (0)	3 (10)	3 (14)	1 (10)	0 (0)

	N = 16	N = 16	N = 1	N = 0	N = 29	N = 21	N = 10	N = 3
SNPs, codon 184								
Y184	2 (12)	10 (63)	1(100)	0 (0)	15 (52)	7 (33)	5 (50)	2 (67)
184Y/F	0 (0)	1(6)	0 (0)	0 (0)	3 (10)	3 (14)	0 (0)	0 (0)
184F	14 (88)	5 (31)	0 (0)	0 (0)	11 (38)	11 (52)	5 (50)	1 (33)

	N = 15	N = 15	N = 1	N = 0	N = 28	N = 19	N = 10	N = 4
SNPs, codon 1246								
D1246	13 (87)	14 (93)	1 (100)	0 (0)	28 (100)	18 (95)	10 (100)	4 (100)
1246D/Y	0 (0)	0 (0)	0 (0)	0 (0)	0 (0)	0 (0)	0 (0)	0 (0)
1246Y	2 (13)	1 (7)	0 (0)	0 (0)	0 (0)	1 (5)	0 (0)	0 (0)

	N = 15	N = 14	N = 1	N = 0	N = 27	N = 18	N = 10	N = 3
Haplotypes†								
NYD	0 (0)	8 (57)	1 (100)	0 (0)	15 (56)	7 (39)	5 (50)	2 (67)
YFD	2 (13)	1 (7)	0 (0)	0 (0)	0 (0)	3 (17)	1 (10)	0 (0)
NFD	10 (67)	4 (29)	0 (0)	0 (0)	12 (44)	11 (61)	4 (40)	1 (33)
NFY	0 (0)	1 (7)	0 (0)	0 (0)	0 (0)	1 (6)	0 (0)	0 (0)
NYY	0 (0)	0 (0)	0 (0)	0 (0)	0 (0)	0 (0)	0 (0)	0 (0)
YYD	1 (7)	1 (7)	0 (0)	0 (0)	3 (11)	3 (17)	0 (0)	0 (0)
YFY	1 (7)	0 (0)	0 (0)	0 (0)	0 (0)	0 (0)	0 (0)	0 (0)
YYY	1 (7)	0 (0)	0 (0)	0 (0)	0 (0)	0 (0)	0 (0)	0 (0)

^†^Each possible haplotype constructed from the mixed infections (wildtype and mutant) is reported; therefore, column totals may sum to > 100%

## Discussion

Both ASAQ and AL remain efficacious for the treatment of uncomplicated falciparum malaria among children in Matanga and Ankazomborona, Madagascar. For both medications, day-28 PCR-corrected efficacy exceeded the 90% threshold below which WHO recommends considering a change in first-line anti-malarials [8]. The findings in the ASAQ arm were consistent with those from the NMCP’s 2012–2016 therapeutic efficacy trials of ASAQ for uncomplicated malaria among individuals of all ages in six sites in Madagascar, including Matanga [9]. Results from that trial, which also followed the WHO protocol, revealed uncorrected and PCR-corrected efficacies above 90% for all six sites. For AL, however, no published studies of efficacy have been performed in Madagascar; thus, the current study serves as a baseline for the country. Of note, the efficacy findings of this study are consistent with ASAQ and AL therapeutic studies conducted in Mozambique, Madagascar’s closest neighbour on the African continent [25, 26].

The Matanga site had a higher rate of re-infection, which could be related to higher transmission intensity there, although in 2016 parasite prevalence among children 6–59 months was estimated at 9% in the zones where Matanga and Ankazomborona are located [18]. Routine health data from 2018 from districts encompassing these two communes suggested significantly higher disease rates among children two months to 14 years of age in Matanga compared with Ankazomborona (163 and 124 per 1000, respectively). In addition to differences between sites, the uncorrected efficacy of AL was lower than that of ASAQ. The shorter half-life of AL compared with ASAQ could indicate a shorter period of post-treatment prophylaxis resulting in higher numbers of re-infections in the AL arms [27, 28]. Additionally, AL absorption may have been compromised in this study because it was not administered with fatty food as recommended by the manufacturer [29]; however, drug levels were not collected, making this assertion impossible to ascertain. Evidence of sub-optimal efficacy of AL has been described in some African countries [10, 11, 30, 31]; reinforcement of correct AL administration (i.e., with fatty food) and close monitoring of efficacy for uncomplicated malaria are warranted in Madagascar.

No *pfk13* mutations associated with artemisinin partial resistance were observed, suggesting continued susceptibility to artemisinin. This is consistent with clinical outcome data, which showed that by day-2, 156/165 (95%) participants in the ASAQ arm and 147/170 (86%) in the AL arm were aparasitaemic and by day-3, all but two participants were aparasitaemic. However, the recent identification of parasites in Rwanda harbouring a *pfk13* mutation, R561H, with associated delayed clearance after treatment with AL underscores the importance of continued monitoring for *pfk13* mutations in African TESs [7].

Mutations in the *pfmdr1* gene have been shown to play a significant role in parasites’ tolerance to some anti-malarials such as chloroquine, amodiaquine, artemisinin derivatives, and lumefantrine; N86 wild type allele is implicated in decreased sensitivity to lumefantrine while the 86Y mutant allele, in combination with mutations in the *pfcrt* gene, is associated with decreased sensitivity to chloroquine and amodiaquine [16, 32, 33]. *pfmdr1* results in this study revealed a high prevalence of the N86 allele (NFD and NYD haplotypes), a finding seen recently in other countries throughout Southern and East Africa [7, 12, 34]. In six sites in Madagascar in 2006, the NFD and NYD haplotypes were observed in only 21.8 and 8.0% samples, respectively, although these frequencies increased to 30.0 and 14.6% just one year later [35]. With 82% of samples in two Madagascar sites containing the N86 allele in this 2018 study, this SNP’s (and associated haplotypes, NFD and NYD) prevalence appears to have increased further, although direct site-to-site comparison is not possible.

No mutations were observed in the 72–76 codons of the *pfcrt* gene, which is consistent with several studies conducted in Madagascar [35, 36]. Mutations in this gene, especially the 76 T codon, were associated with resistance to chloroquine and amodiaquine in several African countries which then reported the “return” of chloroquine-susceptible parasites with wild type alleles, as early as 10 years after chloroquine withdrawal [37, 38]. Madagascar is among countries not recommending the use of chloroquine for treatment of malaria in over 10 years due to high rates of chloroquine treatment failures. In contrast to what was observed in other African countries, chloroquine treatment failures in Madagascar were observed in the absence of *pfcrt* mutations but were significantly associated with the *pfmdr1* 86Y mutant allele [35, 36, 39]. This study is similar to these previous studies, which reported the absence or very low prevalence of *pfcrt* mutations in Madagascar.

## Conclusion

First- and second-line ACT remain efficacious for uncomplicated falciparum malaria in Madagascar; however, periodic monitoring to ensure anti-malarial drug efficacy and the continued absence of worrisome mutations is essential.

## Supplementary Information

**Additional file 1:** Microsatellite lengths of paired Day 0-Day of Failure samples from cases of recurrent parasitemia and a random sample of day 0 samples of non-recurrent parasitemias observed over the 2018 therapeutic efficacy study in Madagascar. Last column shows the posterior probability of recrudescence obtained from Bayesian analysis of observed data.

## Data Availability

The datasets generated and/or analysed during the current study have been de-identified and uploaded to USAID’s data development library (DDL).
